# Estradiol and Progesterone Exhibit Similar Patterns of Hepatic Gene Expression Regulation in the Bovine Model

**DOI:** 10.1371/journal.pone.0073552

**Published:** 2013-09-17

**Authors:** Carla A. Piccinato, Guilherme J. M. Rosa, Alhaji U. N’Jai, Colin R. Jefcoate, Milo C. Wiltbank

**Affiliations:** 1 Endocrinology-Reproductive Physiology Program, University of Wisconsin, Madison, Wisconsin, United States of America; 2 Department of Animal Sciences, and Department of Biostatistics & Medical Informatics, University of Wisconsin, Madison, Wisconsin, United States of America; 3 Department of Pathobiological Sciences and Molecular & Environmental Toxicology, University of Wisconsin, Madison, Wisconsin, United States of America; 4 Department of Pharmacology, University of Wisconsin, Madison, Wisconsin, United States of America; 5 Department of Dairy Science, University of Wisconsin, Madison, Wisconsin, United States of America; University of Navarra School of Medicine and Center for Applied Medical Research (CIMA), Spain

## Abstract

Female sex steroid hormones, estradiol-17β (E2-17β) and progesterone (P4) regulate reproductive function and gene expression in a broad range of tissues. Given the central role of the liver in regulating homeostasis including steroid hormone metabolism, we sought to understand how E2-17β and P4 interact to affect global gene expression in liver. Ovariectomized cows (n = 8) were randomly assigned to 4 treatment groups applied in a replicated Latin Square design: 1) No hormone supplementation, 2) E2-17β treatment (ear implant), 3) P4 treatment (intravaginal inserts), and 4) E2-17β combined with P4. After 14 d of treatment, liver biopsies were collected, allowing 28 d intervals between periods. Changes in gene expression in the liver biopsies were monitored using bovine-specific arrays. Treatment with E2-17β altered expression of 479 genes, P4 472 genes, and combined treatment significantly altered expression of 468 genes. In total, 578 genes exhibited altered expression including a remarkable number (346 genes) that responded similarly to E2-17β, P4, or combined treatment. Additional evidence for similar gene expression actions of E2-17ß and/or P4 were: principal component analysis placed almost every treatment array at a substantial distance from controls; Venn diagrams indicated overall treatment effects for most regulated genes; clustering analysis indicated the two major clusters had all treatments up-regulating (172 genes) or down-regulating (173 genes) expression. Thus, unexpectedly, common biological pathways were regulated by E2-17β and/or P4 in liver. This indicates that the mechanism of action of these steroid hormones in the liver might be either indirect or might occur through non-genomic pathways. This unusual pattern of gene expression in response to steroid hormones is consistent with the idea that there are classical and non-classical tissue-specific responses to steroid hormone actions. Future studies are needed to elucidate putative mechanism(s) responsible for overlapping actions of E2-17β and P4 on the liver transcriptome.

## Introduction

The sex steroid hormones, estradiol-17β (E2-17β) and progesterone (P4), regulate diverse physiological functions including brain function, bone cell metabolism, and almost all aspects of female reproduction through actions on various tissues and cell-types [1], [2]. There is a complex interplay between the actions of E2-17β and P4 in these varied tissues. For example in the uterus, estrogens stimulate uterotropic responses, whereas progestins generally seem to be antagonistic to these estrogen-induced effects [3], [4] **.** Conversely, many of the physiological actions of progestins are dependent upon prior estrogen exposure [5], [6].

To add to the complexity, these two steroid hormones exert their cellular actions through a diverse array of cellular pathways. The classical pathways for steroid hormones involve regulation of gene transcription through binding of activated nuclear receptors to specific steroid response regions of DNA [7]–[10]. Nongenomic actions of steroid hormones have also been described due to binding of E2-17β and P4 to a surprising assortment of membrane-bound receptors [9], [10]. In the liver, many of E2-17β actions may be mediated by estrogen receptor alpha (ERα) which is the predominant form reported in the liver, while subtype beta (ERβ) has not yet been demonstrated [11], [12]. Conversely, there is some controversy about P4 responsiveness and presence/absence of P4 receptor in normal hepatic tissue [13]–[17].

Although numerous studies have evaluated the actions of E2-17β or P4 on global gene expression, the *in vivo* interactions between E2-17β and P4 on global gene expression have only been described in the mammary gland [4], [18], Meibomian gland [19], lacrimal gland [20], and serotonin neurons [21]. Although no studies have reported the *in vivo* interactions of E2-17β and P4 on gene expression in the liver, two studies have demonstrated the effects of these steroids on expression of mRNA in specific cultured hepatic cells. In human hepatoma HuH-7 cells, E2-17β inhibited the proapototic actions of tumor necrosis factor alpha; whereas, P4 blocked E2-17β actions leading to apoptosis [22]. Similarly, rat hepatic stellate cells showed an antagonistic interplay between E2-17β and P4 on a select group of genes regulated by oxidative stress [23]. Thus, these *in vitro* studies are consistent with potential antagonistic actions of E2-17β and P4 in hepatic cells. Given the potential physiological importance of steroid hormone regulation of liver function, particularly steroid hormone metabolism [24], studies evaluating the *in vivo* interactions of female sex steroids on liver gene expression are warranted.

The objective of the present study was to evaluate the effects of E2-17β, P4, and the combination of E2-17β and P4 on the global gene expression profile of the liver using *in vivo* hormone replacement of ovariectomized animals (non-lactating dairy cows) to identify the hepatic genes that were altered by female steroid hormones. Ovariectomy is a classical experimental strategy that provides an animal model of reproductive steroid hormone depletion. Moreover, the bovine model is a valuable model for hormone action in hepatic tissue, since multiple liver samples can be collected from the same animal. Our initial hypothesis was that E2-17β and P4 would produce distinct hepatic gene expression profiles and that P4 would antagonize E2-17β-induced gene expression in the bovine liver. Unexpectedly, we found a remarkable overlap in hepatic gene regulation by E2-17β and P4 treatments.

## Materials and Methods

### Ethics Statement

Animal procedures were approved by the Animal Care Committee of College of Agriculture and Life Sciences at University of Wisconsin-Madison. All surgical procedures were performed under lidocaine anesthesia, and all efforts were made to minimize suffering.

### Animal and Experimental Design

Non-lactating Holstein cows (*Bos Taurus*, n = 8) had their ovaries removed by colpotomy. Treatment was initiated an average of two months after ovariectomy, allowing at least 28 d of recovery for all cows. The experiment was designed as a replicated 4×4 Latin Square. Cows were randomly assigned to sequences of the following four experimental treatments: 1) No hormone treatment - control (CO); 2) E2-17β treatment (E2T, receiving E2-17β ear implant [Compudose implant, 24 mg of E2-17β in a silicone elastomer implant, Elanco Animal Health, Indianapolis, IN]); 3) P4 treatment (P4T, cows had two intravaginal P4 inserts [Eazi-breed CIDR, Pfizer Animal Health]); and 4) E2-17β and P4 treatment (E2P4T, cows had both two intravaginal P4 inserts and an ear implant). Every treatment period lasted for 14 d with replacement of hormone devices after 7 d. After every treatment period, liver biopsies were performed and hormone devices were removed. A 28 d interval was allowed between treatment periods to avoid carry-over effects. To monitor the effectiveness of treatments, blood samples were collected every day around the same time (between 6∶00 and 7∶00 pm; 6 h after feeding).

Cows were maintained at the Dairy Cattle Center – University of Wisconsin, Madison, USA, and a week before the beginning of the trial until the end of the trial were fed to meet maintenance requirements. A total mixed ration (dry cow ration) was prepared following National Research Council [25] recommendations to supply maintenance requirements. Alfalfa silage and corn silage were chosen as forage for the ration and used in the same proportion (50∶50) throughout the trial to avoid variations in macro or micronutrient composition of the diet. Body weight was obtained at the beginning and at the end of each experimental period.

### Hormone Assay

Estradiol-17β concentrations during treatment period were evaluated by E2-17β double-antibody kit (Diagnostic Products Corporation, Los Angeles, CA). Samples were extracted with 4 mL of ethyl ether. The intra-assay coefficient of variation (CV) was 18.9%. Progesterone concentrations from serum samples collected during treatment periods were determined by antibody-coated-tube RIA kit (Diagnostic Products Corporation, Los Angeles, CA) with an intra-assay CV of 10% and an inter-assay CV of 15%.

### Liver Biopsy Procedure

Liver tissue was obtained using a percutaneous biopsy procedure [26] using 12 ml of lidocaine as local anesthetic. Once collected, tissue was snap-frozen in liquid nitrogen, and stored at −80°C for further analysis.

### Messenger RNA Isolation and Microarray Analysis

The microarray experiment was performed using the Affymetrix Bovine GeneChip containing 24,072 probesets, representing over 23,000 transcripts and 19,000 UniGene clusters. Samples from each cow in each period/treatment were individually assayed in independent microarry chips. Messenger RNA was isolated from the liver samples using Magnetight oligo (dT) magnetic beads (Novagen, Madison, WI). Quantity and quality of mRNA was assessed using a spectrophotometer (Nanodrop-1000) and the Agilent 2100 BioAnalyzer (for mRNA integrity using microfluidic analysis). Messenger RNA samples (2 µg) that passed the quality control steps were biotin-labeled with Message Amp II Biotin Enhanced kit (Ambion, Inc, CA) following the manufacturer’s protocol. The Affymetrix poly-A RNA control kit was spiked into samples to serve as a control for variation in labeling and hybridization of samples. After purification, RNA fragmentation was accomplished by heating biotinylated antisense RNA to 94°C for 35 min in a MgCl_2_ buffer. Fragmented and unfragmented RNA (1 µg) were analyzed on a 2% agarose gel to assure that most fragments were below 150 bases.

After purification of the labeled RNA, an array hybridization procedure was performed at the Gene Expression Center of the University of Wisconsin-Madison Biotechnology Center. The fragmented and biotinylated copy RNA was hybridized to the Bovine GeneChip expression arrays for 16 h at 45°C, according to the manufacturer’s instructions. Arrays were processed using the Affymetrix Fluidics 450 Station and images were captured using an Affymetrix GC3000 scanner. Array data were captured using the Affymetrix GCOS 1.4 software. Fluorescent signals corresponding to hybridization intensities were analyzed with the Affymetrix Microarray Suite (MAS) 5.0 algorithm using default analysis settings.

### Statistical Analysis

Data acquisition and normalization was done using Affymetrix MAS 5.0 software. Careful evaluation was made when summarizing image analyses into expression indices for each gene in each slide, as well as preprocessing the data to remove possible sources of systematic, non-biological variation. Only genes flagged as present by MAS 5.0 in at least one array were used in the statistical analysis.

Genes flagged as present were log transformed to facilitate normalization requirements. The log-transformed data were then analyzed for detection of differentially expressed genes between the four treatment groups (CO, E2T, P4T, and E2P4T). This analysis was performed using a mixed model analysis of variance (ANOVA) approach for a Latin square design [27]. The following gene-specific model was utilized:
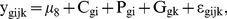
(1)


where: y_gijk_ represents the normalized log intensity signal, µ_8_ is the overall mean for gene g, C_gi_ is the random effect of cow i, P_gi_ is the random effect of period j, G_gk_ is the fixed effect of the experimental group k, and ε_gijk_ is a residual term. The index g in each of the model components indicates that those effects are specific for gene g. To complement each gene-specific ANOVA, contrasts involving the experimental groups were used to test the main effects of E2-17β and P4, as well as the interaction between them. These analyses were performed using the MIXED procedure of SAS (SAS, 2004). In order to account for the multiple testing problem, the p-values from the ANOVA-F tests were converted to q-values [28] to establish statistical significance based on a false discovery rate (FDR).

### Data Filtering and Annotation

First, differentially expressed genes identified by ANOVA were ranked and prioritized using a *P*-value cut-off <0.01 to identify an initial subset of differentially expressed genes for further investigation. From this subset of genes, we next performed a contrast analysis in order to identify genes that were differentially expressed between specific treatment groups by selecting for genes with 1.25 absolute fold-change in addition to *P*-values <0.05. These same combined criteria were also used to examine overlapping differentially-regulated genes (genes that were differentially expressed by different treatments).

Genes that matched the criteria were subsequently analyzed using principal component analysis (PCA) and hierarchical clustering using SAS, with Euclidian distance to group data points with the smallest distances.

Gene function and identification was achieved by the NetAffyx (www.affymetrix.com) based on the Gene Ontology database, followed by a study of overrepresented lists of genes using DAVID (http://david.abcc.ncifcrf.gov), in which a chi-squared based test was used to detect overrepresented gene categories on the lists of differentially expressed genes [29]. Because of limitations on bovine genome annotation, only 79% of differently-expressed genes could be further annotated by DAVID. All the microarray data is MIAME compliant and the raw data have been deposited in a MIAME compliant database, the National Center for Biotechnology Information Gene Expression Omnibus (www.ncbi.nlm.nih.gov/gsd, accession number: GSE45857).

## Results

### Circulating Concentrations of Steroid Hormones

**Figure 1:**
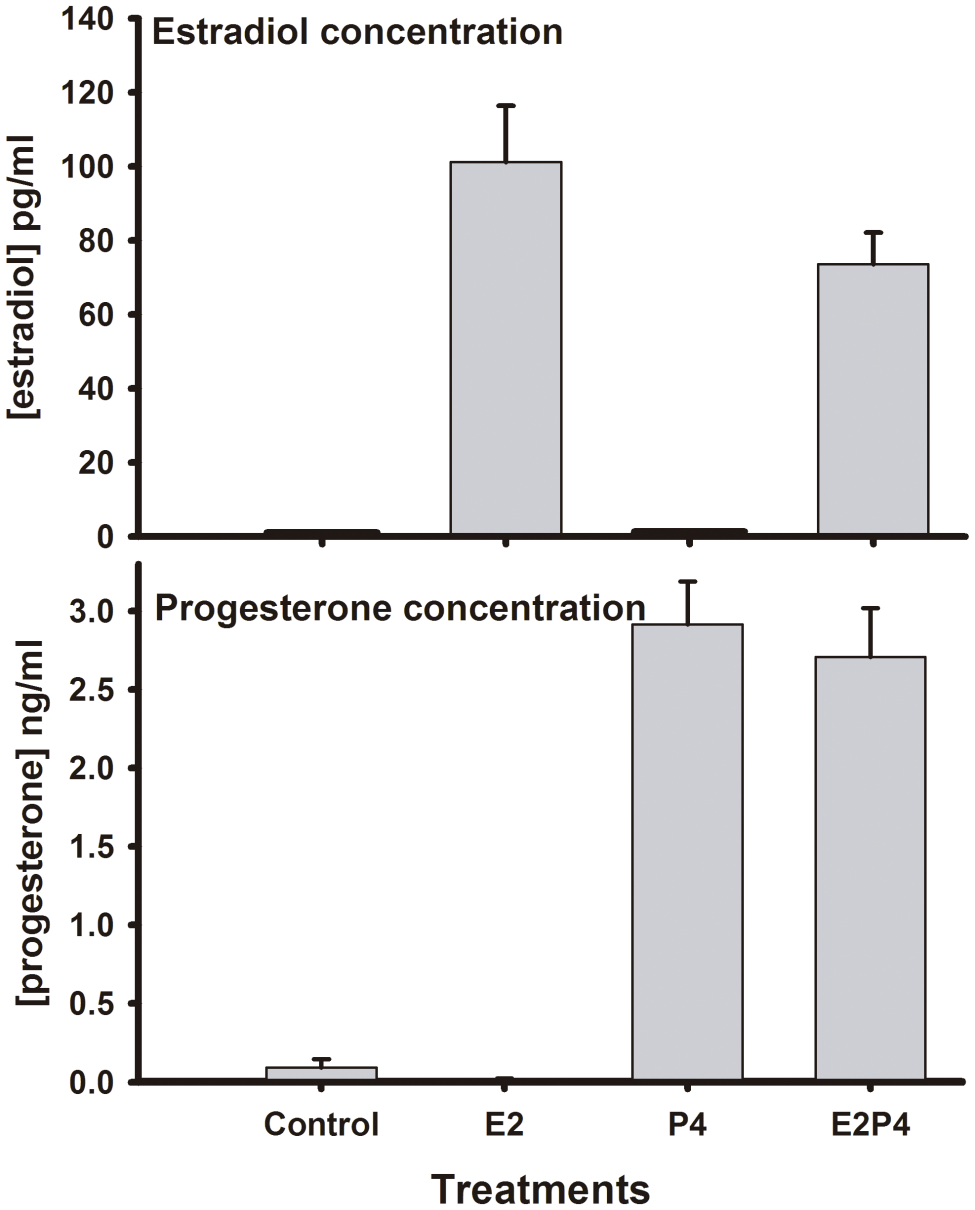
Circulating concentrations of steroid hormones. The daily concentration of estradiol (pg/ml) and progesterone (ng/ml) during hormonal treatment was averaged in this plot (n = 8).

Cows were ovariectomized at least 28 d prior to the beginning of the trial and then assigned to hormonal treatments for periods of 14 d separated by treatment rest intervals. Basal concentrations of E2-17β and P4 were extremely low in all ovariectomized cows used in this experiment and much lower than the cows treated with E2-17β or P4 (overall, *P*<0.01). During the first 24 h of implant insertion, a peak of each steroid hormone was observed at around 12 h (data not shown). Figure 1 shows the mean circulating concentration of steroid hormones during the days of hormonal treatments. Treatment with E2-17β ear implants increased (*P*<0.05) circulating E2-17β concentrations to 101.1±15.2 pg/mL (E2T) and 73.6±8.4 pg/mL (E2P4T), while CO (0.98±0.1 pg/mL) and P4T (1.2±0.1 pg/mL) did not elevate E2-17β concentrations. Treatment with P4 (using CIDR) increased (*P*<0.01) circulating P4 to 2.9±0.3 ng/mL (P4T) and 2.7±0.3 ng/mL (E2P4T). There were very low concentrations of circulating P4 in cows from CO (0.09±0.05 ng/mL) and E2T (0.01±0.01 ng/mL) groups (these values were close to P4 assay sensitivity). Thus, all treatments were effective in increasing circulating steroid hormones. In addition, cows from all treatment groups had similar body weights (613±39 kg) that only increased marginally during the entire experiment (28.6 kg of weight gain on average), with no differences between treatment periods.

### Overall Hepatic Gene Expression Results

**Figure 2:**
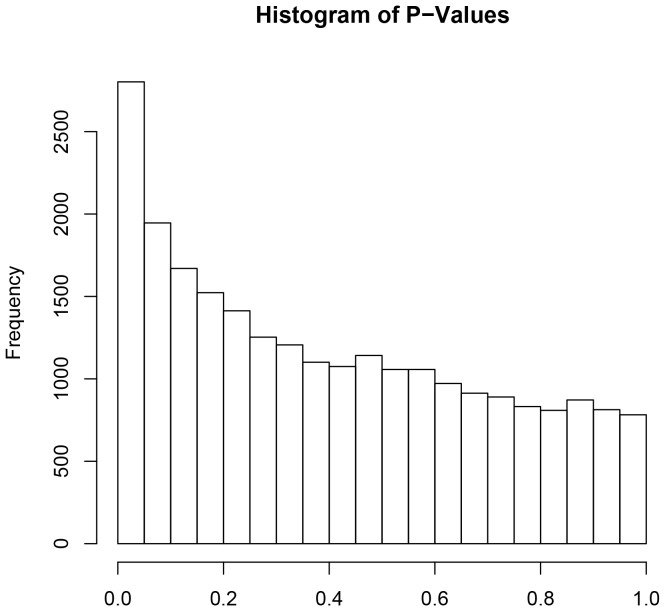
Histogram of *P*-Values. Using the ANOVA analysis, a histogram of P-values was generated based on the treatment effects for genes present in at least one array (18,603 genes). It is observed a higher than expected (under the null hypothesis of no treatment effects) number of genes with low *P*-Values.

An evaluation of signal values from each microarray indicated that two of the arrays produced signals that were significant outliers (one from P4T and another from E2P4T) compared to the other arrays. These arrays were not utilized in subsequent analyses. At the end, 8 arrays in the E2T and in the CO, and 7 arrays in the P4T and E2P4T were further analyzed. Figure 2 shows a histogram of *P*-values for differences between treatments for each of the genes flagged present by MAS 5.0. Under the null hypothesis of no treatment effect on any gene, it is expected that a similar number of genes would be found within each of the 20 histogram categories (∼930 genes per category). However, as shown, there are only about 800 genes in the higher *P*-value categories and an increasing number in the lower *P*-Value categories. This indicates that differential expression between treatments was occurring for many genes. In particular, there are more than 2000 genes in the *P*<0.05 category and these genes were used for subsequent selection of differentially expressed genes, as described in the Materials and Methods. Following the ANOVA, 592 genes were determined to have significant (*P*<0.01) treatment differences in steady-state concentrations of mRNA and these genes were utilized in subsequent analyses. This represented 3.2% of the total number of genes that were found to be present in the bovine liver (18,703 genes). After evaluation of q-values for those 592 differentially expressed genes, the false discovery rate was determined to be 21%.

**Figure 3:**
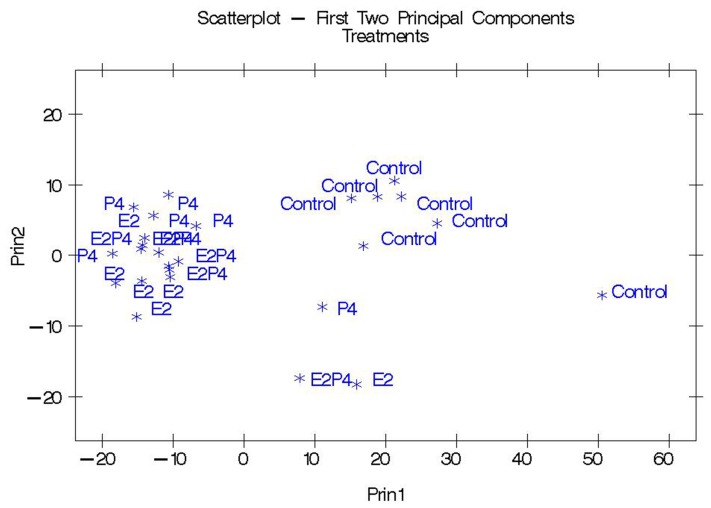
Principal component analysis. Principal component analysis (PCA) using gene expression measurements from 592 genes selected based on significance level (*P*<0.01). Control samples (CO) clustered effectively separated from the estradiol-17β (E2), progesterone (P4) and estradiol-17β+progesterone (E2P4T) treated samples.

Principal component analysis was next performed, based on the concentrations of the 592 differentially expressed genes in the microarray analysis. Each individual liver biopsy was analyzed for the two principal components and the scatter-plot for each array in the four treatments is shown in Figure 3. Gene expression in the CO arrays was found to be quite distinct from gene expression in any of the other treatment groups, shown by the tight cluster of CO arrays. In contrast, the great majority of the samples from the three treatment groups were clustered together and apart from the CO cluster. One sample from each of the groups was easily distinguished from the two main clusters.

**Figure 4:**
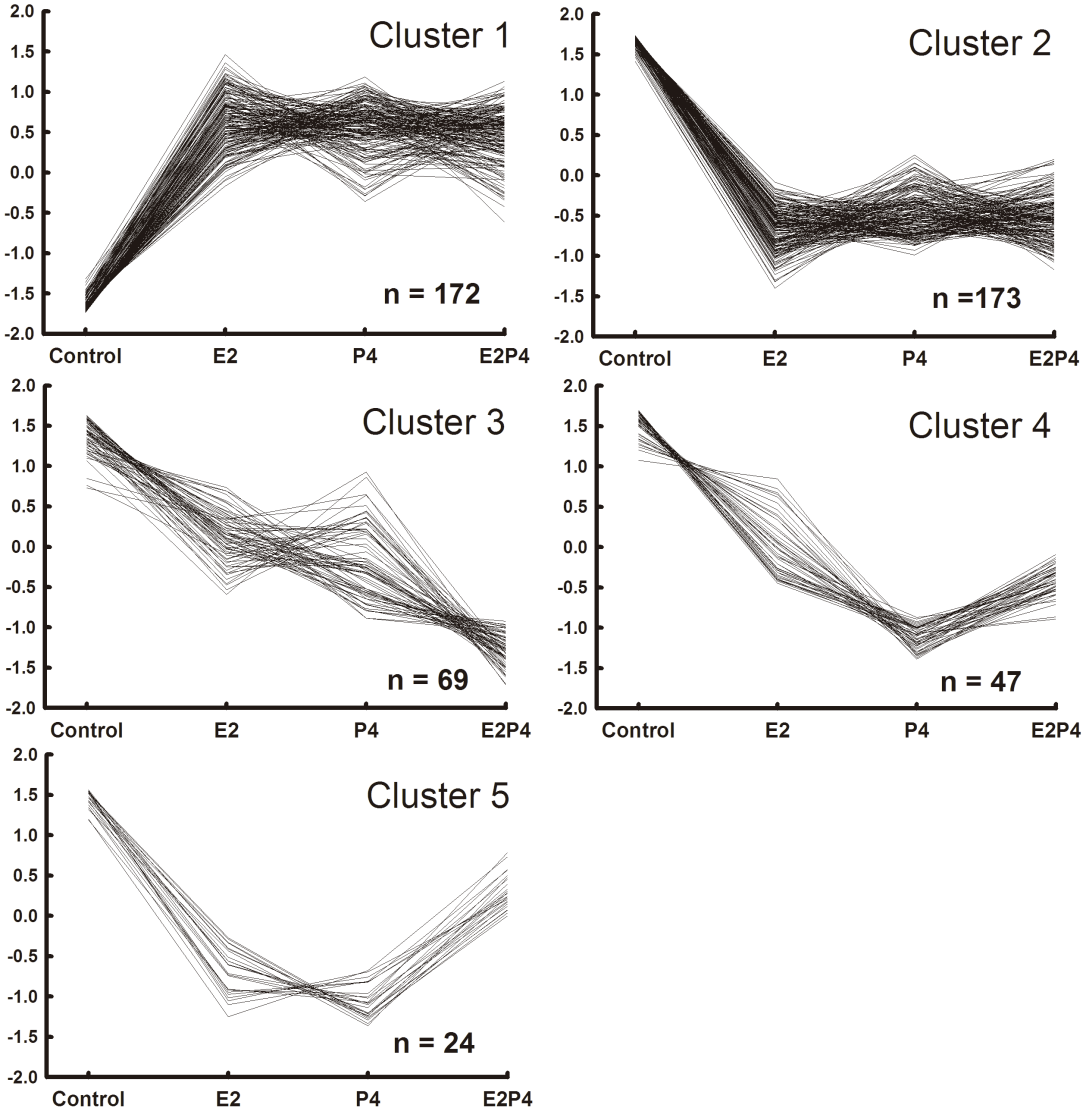
Hierarchical clustering analysis of significant genes by treatment. Unsupervised hierarchical clustering analysis produced five main clusters. The clusters correspond to upregulated genes in all treatments (Cluster 1, n = 172), downregulated genes in all treatments (Cluster 2, n = 173), synergized downregulated genes (Cluster 3, n = 69), marked P4 downregulated genes that were moderately downregulated by estradiol-17β and estradiol-17β+progesterone (Cluster 4, n = 47), and genes that were downregulated by estradiol-17β and progesterone alone and antagonized by the combined treatment (Cluster 5, n = 24). An extensive similarity of magnitude and direction of expression can be observed between significantly regulated genes.

Cluster analysis of expression patterns for individual genes between treatments was next performed for the 592 differentially-expressed genes in order to determine whether there were common patterns of treatment effects for different genes. The clusters were classified according to the pattern of gene expression for each individual gene among the treatments. A complementary analysis was performed to verify the percentage of genes that were significantly altered by each treatment among the genes indicated in each of the cluster patterns. For the current cluster analysis, only genes with fold changes greater than 1.25 and *P*-values <0.05 in a given contrast between treatments were considered significant. There were 20 clusters generated by this analysis and the majority of the differentially expressed genes (485 genes = 82%) fit into 5 main clusters. The remaining 107 genes were categorized into 15 other less-representative clusters. Figure 4 depicts the expression pattern for the genes that fit into the 5 main clusters. The number of genes that fit into each cluster was 172, 173, 69, 47, and 24 genes, for clusters 1 to 5 respectively.

The two major clusters (1 and 2) found gene expression levels in all three treatment groups clearly distinct from gene expression in CO. Cluster 1 contained genes that were up-regulated similarly by E2T, P4T, and E2P4T. Of the 172 transcripts in cluster 1, a total of 121 of these transcripts (70.3%) were significantly upregulated in all treatments when compared to CO. Conversely but analogously, cluster 2 contained genes that were down-regulated by all of the treatments. Of the 173 genes contained in cluster 2, a total of 160 genes (92.5%) were significantly down-regulated by all treatments compared to CO. The majority (58.3%) of genes were grouped into these first 2 clusters. Cluster 3 is characterized by a synergism between E2-17β and P4, since the levels of expression of these transcripts were lower in E2P4T than in E2T or P4T alone. A total of 22 of the 39 genes (56.5%) that were classified into cluster 3 showed a significantly decreased gene expression for E2T and P4T compared to CO and for E2P4T compared to E2T or P4T alone. Cluster 4 contained genes that were most markedly down-regulated by P4T and least down-regulated by E2T. Out of 47 genes clustered in cluster 4, there were 29 genes (61.7%) that were significantly down-regulated by all treatments, compared to CO. Cluster 5 contained genes that were down-regulated by E2T or P4T alone but were less down-regulated by E2P4T. Of the 24 transcripts classified into cluster 5, there were 17 transcripts (70.8%) that were significantly decreased by E2T or P4T; whereas, the pattern produced by E2P4T did not differ from the CO pattern. Examples of genes that were classified into clusters 1–5 are listed in Table 1. Clearly the expression patterns for genes in cluster 1 are distinct from the other 4 clusters since these genes are up-regulated. However, various patterns of decreased gene expression for all treatment groups are evident for genes in clusters 2–5.

**Table 1:** Examples of genes significantly regulated by estradiol-17β (E2T), progesterone (P4T) and estradiol-17β+progesterone (E2P4T) treatment, grouped in 5 distinct clusters.

Gene	Gene Description	Cluster	E2T		P4T		E2P4T	
			Fold-Change	*P*	Fold-Change	*P*	Fold-Change	*P*
*SECTM1*	secreted and transmembrane 1	1	5.6	4.6E-05	4.0	4.8E-04	2.4	1.2E-02
*PPP2R5E*	protein phosphatase 2, regulatory subunit B′, epsilon isoform	1	3.9	1.4E-03	2.7	1.4E-02	3.6	3.0E-03
*CDK2*	cyclin-dependent kinase 2	1	2.5	2.2E-03	2.0	2.1E-02	3.0	1.0E-03
*MGC165862*	hypothetical LOC614805	1	2.2	8.9E-05	2.0	4.2E-04	1.5	2.1E-02
*C3orf57*	hypothetical protein LOC780785	1	1.9	8.7E-03	2.3	3.1E-03	2.2	4.2E-03
*ADRB3*	adrenergic, beta-3-, receptor	2	−3.7	7.5E-05	−3.9	8.2E-05	−2.8	8.3E-04
*CORO2A*	coronin, actin binding protein, 2A	2	−3.5	1.9E-04	−2.7	2.1E-03	−1.9	2.7E-02
*AP4M1*	adaptor-related protein complex 4, mu 1 subunit	2	−3.5	1.2E-04	−2.6	1.7E-03	−2.5	2.5E-03
*SALL2*	sal-like 2 (Drosophila)	2	−2.9	7.8E-04	−2.1	1.1E-02	−2.7	1.8E-03
*CCDC97*	Coiled-coil domain containing 97	2	−2.8	8.3E-03	−3.0	7.3E-03	−3.6	2.9E-03
*EHBP1L1*	EH domain binding protein 1-like 1	3	−2.1	2.5E-02	−2.9	4.5E-03	−3.7	1.1E-03
*HBB///HBE1*	hemoglobin, beta///hemoglobin, epsilon 1	3	−2.3	3.4E-03	−2.2	6.4E-03	−3.4	2.5E-04
*TMEM179B*	Transmembrane protein 179B	3	−2.0	9.8E-03	−1.8	2.7E-02	−2.8	1.1E-03
*FBXO15*	F-box protein 15	3	−1.7	1.8E-03	−1.7	2.9E-03	−2.3	5.2E-05
*HIP1R*	huntingtin interacting protein 1 related	3	−1.7	4.9E-03	−1.5	3.6E-02	−2.3	3.6E-04
*TRPC2*	transient receptor potential channel 2	4	−2.9	3.2E-02	−7.5	1.1E-03	−3.8	1.5E-02
*NAP1L4*	Nucleosome assembly protein 1-like 4	4	−2.8	1.3E-02	−5.8	4.1E-04	−3.5	4.6E-03
*ERCC3*	Excision repair cross-complementing rodent repairdeficiency, CC 3	4	−2.0	8.6E-03	−2.9	5.0E-04	−2.1	6.5E-03
*LOC514916*	similar to Dual-specificity tyrosine-(Y)-phosphorylationregulated kinase 2	4	−1.7	1.6E-05	−2.0	1.7E-06	−1.8	1.4E-05
*LOC526608*	similar to endo-beta-N-acetylglucosaminidase		−1.4	1.2E-02	−1.9	2.6E-04	−1.7	1.0E-03
*VPS53*	vacuolar protein sorting 53 homolog (S. cerevisiae)	5	−1.7	6.0E-03	−2.1	7.1E-04	−1.5	3.4E-02
*COL22A1*	Collagen, type XXII, alpha 1	5	−1.7	6.4E-04	−1.6	2.2E-03	−1.3	5.0E-02
*LOC506005*	similar to connexin43-interacting protein of 150 kDa	5	−1.6	9.2E-03	−1.9	1.5E-03	−1.4	4.8E-02
*ZNF527*	zinc finger protein 527	5	−1.4	6.3E-03	−1.6	4.3E-04	−1.3	4.0E-02
*PI4KA*	phosphatidylinositol 4-kinase, catalytic, alpha	5	−1.3	4.2E-03	−1.6	2.2E-04	−1.3	1.7E-02

### Quantitative Analysis of Gene Expression Responses Induced by each Individual Treatment

**Figure 5:**
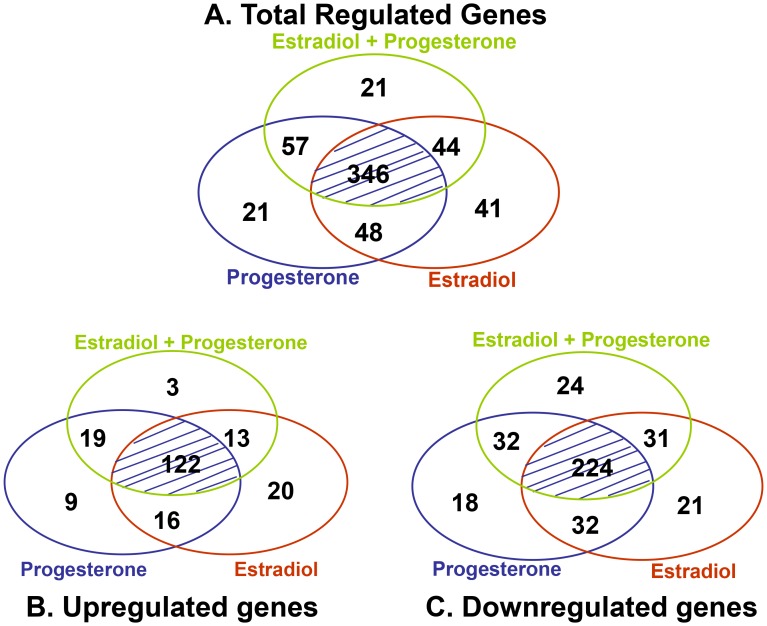
Venn diagrams. Venn diagrams of all genes regulated by estradiol-17β (E2T), progesterone (P4T) and the combined treatment (E2P4T) (A); genes up-regulated (B) and down-regulated (C) by all treatments; the highlighted intersection represents genes that were commonly regulated by all treatments. Each of the circles represents a different treatment. The numbers in the intersection of the circles represent the number of transcripts that were affected in the treatments represented by those respective circles. The numbers in the outer portion of each circle represent the number of transcripts that were exclusively affected in the treatment represented by that particular circle. There was a total of 578 differentially expressed genes (A); 202 upregulated genes (B) and 382 downregulated genes (C) compared to controls, considering the following stringency: fold change >1.25 and *P*<0.05.

To discriminate the effects induced by E2T, P4T and E2P4T, a subsequent analysis was performed that utilized the genes that were below the *P*<0.01 cut-off and also had a minimum of a 1.25-fold change in expression. Using these criteria, a total of 578 differentially expressed genes (97.6% of the selected 592 genes) were detected and the distribution of this differential expression by treatment group is shown in the Venn Diagrams in Figure 5. Among those 578 genes, E2T altered expression of 479 genes, P4T altered expression of 472 genes, and E2P4T altered expression of 468 genes. Comparison of differentially expressed genes between all treatments revealed 346 genes (60%) that were similarly altered in expression by all three treatments (Figure 5A). Most of the genes that were significantly altered by one of the treatments were also altered by at least one other treatment (495 genes; 85.6%). There were 149 genes that were significantly altered by only two of the treatments (Figure 5A). Interestingly, a similar distribution of differential gene expression was found for up-regulated genes (Figure 5B) and down-regulated genes (Figure 5C). However, there were more genes that were down-regulated by at least one of the treatments (382 of 578 genes; 66%) than genes that were up-regulated by at least one of the treatments (202 of 578 genes; 35%). Figure 5A also shows that a relatively small number of genes (n = 83) were altered by only one of the treatments and not by any other treatment with numerically more of these genes altered by E2T (41 genes) than P4T (21 genes) or E2P4T (21 genes). Note that only 6 genes had significant down-regulation by one treatment and up-regulation by a different treatment (Figure 5A and B).

### Functional Analysis of E2T, P4T, and E2P4T Gene Responses

To understand the functional relevance of the differentially expressed genes induced by steroid hormone treatments, we performed functional annotation analysis. Treatment with E2-17β altered expression of genes associated with oxidative phosphorylation, respiratory chain, mitochondrial membrane, and ion trans-membrane transport activity in the bovine liver. These were also the top enriched pathways detected for P4T and E2P4T. All of these gene clusters had enrichment scores (z-scores) greater than 3.0 and high significance (*P*<0.0001) for all treatments. Genes up-regulated by all treatments (cluster 1) were enriched in genes involved with oxidative phosphorylation (z-score: 8.73), mitochondrial function (z-score: 8.4), and inorganic trans-membrane transporter activity (z-score: 5.4). Genes that were commonly down-regulated by all treatments (cluster 2) were related to ion binding processes, but with a lower z-score (1.81).

Considering all 479 genes that were regulated in E2-treated cows, 171 were up-regulated and 308 were down-regulated. The top 20 up-regulated and down-regulated genes are shown in Table S1. Examples of genes that were up-regulated by E2-17β include: *SECTM1*, protein phosphatase 2-regulatory subunit B, cyclin-dependent kinase 2, and *EGF*-containing fibulin-like extracellular matrix protein 2 (EFEMP2). Most of the genes, except EFEMP2 had similar changes in expression in response to the other treatments. Examples of genes that were down-regulated by E2-17β are such as: G-protein-coupled receptor 155, collagen type II alpha 1, peroxisome proliferator-activated receptor delta, insulin-like growth factor binding protein 2, and 2 zinc finger related proteins: zinc finger *MYND*-type containing 15 and zinc finger/*SCAN* domain containing 29. Again, other treatments generally had similar direction of effects although magnitude and significance were generally less than for E2T.

Among the genes that were regulated in P4-treated cows, 166 were upregulated and 306 were downregulated. The top regulated genes in P4-treated cows are listed in Table S2. As can be seen, some of the same genes are found on this list as on the list of top 20 E2-17β-regulated genes. Other genes that were substantially up-regulated by P4T include: gap junction protein delta 2, pyruvate dehydrogenase (lipoamide) beta, nucleosome assembly protein like-5, and organic solute transporter alpha-like. Cytochrome *P450 2C87*, *FAT* tumor suppressor homolog 1, and *S100* calcium-binding protein A10 are examples of genes differentially up-regulated by P4 and not by the other treatments.

Cows treated with both E2-17β and P4 produced 157 up-regulated and 311 down-regulated genes (examples in Table S3). All genes exclusively up-regulated in E2-17β and P4 treated cows using our stringency criteria are not yet identified and annotated (transcribed locus). Some examples of genes that were exclusively down-regulated by E2P4T include insulin receptor, atrophin-1, neurochondrin, and glucocorticoid modulatory element binding protein 2.

## Discussion

The present study explored the interplay between E2-17β and P4 specifically in the liver employing global gene expression by microarray analysis to identify patterns and networks of genes regulated by E2-17β and/or P4. Although not considered a classical tissue for steroid hormone action, the liver has been previously found to respond to estrogens with changes in gene expression [12], [30]. Indeed, appreciable effects of both steroid hormones are evident in the present study. Identification and characterization of hepatic genes that are regulated in E2-17β, P4, and E2-17β plus P4 treated cows is an important step toward understanding the mechanism of action of estrogenic and progestogenic compounds in physiological and pathological conditions in liver cells. In addition, the central role of liver in controlling the biological activity and circulating concentration of steroid hormones make the potential interplay of steroids with this tissue of particular interest to endocrinologists. After completing this research, the most striking and surprising finding was that gene regulation was so similar in the liver of cows treated with either E2-17β, P4, or both steroid hormones since these steroid hormones are generally thought to have distinct receptor activity and biological actions in most tissues.

The similarity of gene expression changes produced by these two hormone treatments separately or together was substantial both with respect to the genes stimulated and suppressed and to the magnitude of the responses. First, principal component analysis demonstrated strong similarity between the steroid treatments groups with almost every treatment array placed a substantial distance from the CO arrays. This result was mostly due to distinctive differences between the CO arrays and arrays for any of the treatment groups for values derived for Principal Component 1. The Venn diagrams represent a second type of evidence that E2-17β and P4 had similar actions on hepatic gene expression. Clearly, the majority of genes were regulated in all three treatment groups (346 of 578 = 60%) with only 14% (83/578) of genes regulated by only one of the treatment groups. This overlapping of genes that were regulated in all three treatment groups was observed for genes that were either up-regulated or down-regulated by the treatments. Thirdly, the clustering analysis also demonstrated the remarkable similarity in magnitude and direction of hepatic gene regulation by animals treated with either P4 or E2-17β, with the majority of regulated genes grouped into cluster 1 (172 genes), with genes similarly up-regulated by all three treatments, or cluster 2, with genes similarly down-regulated in all three treatments (173 genes). A perusal of any of the supplementary tables for the top genes regulated by any particular treatment shows that most of these genes were also similarly regulated by the other 2 treatments and that these similar effects were usually statistically significant for all 3 treatments. Thus, we are left with the clear conclusion that overall gene expression in the liver was similarly regulated by 14 days of treatment with E2-17β, P4, or E2-17β plus P4. These remarkable results are even more noteworthy given that the experiment utilized a Latin-square design to account for individual variability and therefore each individual animal had a liver biopsy followed by a microarray analysis in each of the experimental treatment groups. The replication of multiple treatment groups in the same animals, with a sizeable number of animals for a microarray experiment, makes the results of this experiment particularly powerful, from a statistical viewpoint. Other studies have also noted some similarities between E2-17β - and P4-induced gene expression for the lacrimal gland [20] and Meibomian Gland [19], although not to the same degree as noted in our study. Using a similar approach to understand the patterns of regulation of steroid hormones in the bovine endometrium, Shimizu and collegues [31] found that treatment with E2-17β alone, or with P4 followed by increased E2-17β (P4+E2), produced similar patterns of gene expression change that differed substantially from gene expression in the endometrium of cows treated with only P4. There were surprisingly few genes (20 out of a total of 1291 differentially expressed genes) that were common between all three treatment groups. Thus, in classical steroid responsive tissues the pattern of gene expression regulation by E2-17β and P4 are very distinct from the highly similar regulatory changes that were found in our studies in liver.

The near identity of clusters 1 and 2 responses to E2-17β and P4 and their complete lack of additivity, suggests that liver gene expression is regulated through pathways that are similarly controlled by these two steroids and may be maximally activated by the doses of either steroid that were used in this study. This represents the complete opposite of our initial hypothesis that E2-17β and P4 would have distinct actions or possibly antagonistic actions on gene expression in the bovine liver. This hypothesis was based on previous results showing distinct biological responses and distinct patterns of gene expression for E2-17β and P4 actions in a variety of tissues, for example in the endometrium [4], [31], [32], [33]. In our study, opposite effects of E2-17β and P4 were detected in clusters 6 and 7 (not shown), but very few genes were found in these clusters (total of 16 genes). Antagonistic effects of E2-17β and P4 have been described in cultured hepatic stellate cells exposed to oxidative stress [23]. In the bovine endometrium, there were very distinct patterns of gene expression regulated by the two hormones. For example, there were over 300 genes that were down-regulated by E2-17β but were up-regulated by P4 [31]. In contrast to our study, the majority of steroid-responsive genes were regulated differently by E2-17β than by P4. In endometrial adenocarcinoma cells, the estrogenic metabolite tibolone elicited a distinct set of regulated genes, compared to its progestogenic metabolite with only 13% of genes demonstrating coregulation (the great majority presented opposite effects) [34]. The authors of that manuscript indicated that estrogenic and progestogenic properties of tibolone regulate similar biological processes but through regulation of distinct genes. In the present study, the functional annotation data also indicated that similar biological processes were regulated by the two steroids in liver but in contrast to their results we observed almost identical genes regulated by E2T, P4T, and E2P4T. Some genes that were regulated by E2T have been previously reported [31], [35], such as Cyclin-dependent kinase 2 (*CDK2*), collagen type II alpha 1, and *UDP*-Gal:betaGlcNAc beta 1,4- galactosyltransferase, polypeptide 1 (*B4GALT1*). Other genes that we identified in this study have not been previously described such as myopalladin and G protein-coupled receptor 155 even though other G protein-coupled receptors had already been shown to be involved in the rapid actions of E2-17β [36], [37]. The distinct down-regulation of genes by E2-17β in our experiment was similar to the robust down-regulation by E2-17β of global gene expression in breast cancer cells [38] and the bovine mammary gland [35], [39]. Similarly, the combination of E2-17β and P4 induced predominantly down-regulation of genes involved in the neuroplasticity of the raphe region [21]. In the bovine endometrium, P4 primarily up-regulated gene expression, whereas, E2-17β produced similar numbers of down-regulated and up-regulated genes [31]. Progesterone alone or combined with E2-17β down-regulated a large number of genes in the lacrimal gland [20] and in the Meibomian gland [19]. Thus, although somewhat unexpected results were found in our study, the results are not entirely inconsistent with results that have been previously reported for E2-17β and P4 regulation of gene expression.

It is not clear whether our E2-17β results are due to regulation of the classical ER through the estrogen-response element [2] or by other mechanisms. Although *ERα* and *ERβ* were present on the Affymetrix bovine array, their transcripts were almost undetectable in our tissues. Estrogen receptor *α* expression has been previously described in the rodent liver [11], [40], [41]. However, expression of *ERα* in hepatic tissues is very low when compared to the uterus, pituitary, ovary, kidney, and adrenal gland [11]. The rodent liver is highly responsive to an antagonist of both ERα and ERβ despite low or absent levels of these receptors in the liver [41]. This suggests that mechanisms of estrogen actions in the liver could involve indirect mechanisms through other signaling pathways or even other tissues. Non-genomic pathways regulated by E2-17β have been described in bone, mammary, vasculature, and nervous tissues [36], [42]. Previous studies have not carefully evaluated the non-genomic actions of E2-17β in the liver although one study reported that E2-17β reduced hepatic injury after trauma-hemorrhage through the PKA-dependent pathway via the membrane G protein-couple receptor 30 in a mechanism that is independent of *ERα.* [37].

Similarly, it seems unlikely that the classical pathway of P4 binding to PR and regulating P4-response elements is the underlying explanation for the changes in gene expression observed after P4 treatment. The *PR* was also represented in the Affymetrix Bovine Gene Chip. Progesterone receptor transcripts were also very low and flagged as present in only ∼ 30% of the arrays of liver samples. Previous studies have failed to find *PR* expression in normal liver tissue [15], [16]; although *PR* has been identified in certain hepatic abnormalities [15], [23]. There are other non-classical P4 receptors [9], [10] that may mediate some of the progesterone actions in liver [9], [17], such as the progesterone receptor component 1 (PGRMC1) that we detected as present in all our microarrays.

Nevertheless, even if a clear presence of steroid receptors was detected in the liver, it would still seem unlikely that the presence of estrogen and P4-response elements in the same gene promoters could account for the level of similarity in gene expression results described here. An alternative explanation is that P4 may have an estrogenic effect in the liver. It is possible that P4 could be converted to androgens peripherally [43], [44] or in the liver [45] and then aromatized to estrogen; although, the bovine P45017*α* enzyme does not appear to catalyze this conversion [46]. In addition, aromatase has not been detected in normal liver [45] and was not detectable in our hepatic samples in this study. Another possible site for conversion of androgens to estrogens is peripheral and visceral fat tissue [44]. Nevertheless, circulating E2-17β concentrations remained undetectable in the P4-treated animals suggesting that this explanation is not plausible or that small increases in local concentrations of E2-17β were sufficient to exert substantial and similar biological actions as observed for E2T. Thus, this technical explanation, although implausible, cannot be completely discounted.

Since neither steroid significantly increased circulating levels of the other, we advance the hypothesis that these common liver gene expression changes arise from signaling processes that are shared by estrogen and progesterone receptors (classical or non-classical), either within the liver or, more likely, from sites in external tissues. Thus, an alternative mechanism is that E2-17β and P4 are regulating endocrine pathways or neural signaling from outside the liver. Such pathways may be producing the common actions of these steroids in the liver. Indeed, the liver is sensitive to regulation through the sympathetic nervous system through sinusoidal release of catecholamines and neuropeptide Y. The liver is regulated by numerous hormones such as: insulin, glucagon (from pancreas), cortisol (from adrenal glands), and growth hormone (from pituitary) and each of these hormones is regulated by complex endocrine and neural pathways. An attractive candidate in this context could be growth hormone since it has clear actions in the liver and has been shown to be differentially regulated by kisspeptin-10 in the presence of E2-17β or P4 [47] . In that study, growth hormone was increased by kisspeptin-10 treatment in ovariectomized cows that were treated with either E2-17β or P4 but growth hormone was not increased by kisspeptin-10 in the absence of E2-17β and P4. In addition, E2-17β has been found to increase circulating growth hormone concentrations and insulin-like growth factor-1, in addition to increasing the mRNA for their receptors in the liver [48].

Another proposed mechanism to explain the similarity in gene expression for E2-17β and P4 is that both steroids or even their metabolites could be binding to other members of the steroid-hormone receptor superfamily such as pregnane X receptor [49] or constitutive androstane receptor [49], [50], eliciting activation of common pathways. One example is the low affinity of E2-17β for other nuclear receptors such as the androgen receptor [51].

A final possible explanation is that E2-17β and P4 might be eliciting similar protein-protein or nucleic acid-protein interactions to modulate gene expression without directly binding to DNA [2]. This can occur at the post-transcriptional regulation level, with rates of degradation of specific mRNA regulated by *cis*-elements and *trans*-acting factors. Similar regulation of mRNA stability by different steroid hormones has been well-described (for review see [52]) and this is a potent and important action of steroid hormones [52]. It seems plausible, although not yet tested in any tissue that E2-17β and P4 may have similar actions in regulation of mRNA stability in the liver or other tissues and this could be the common mechanism that produces the unexpected similarity in E2-17β and P4 regulation of hepatic gene expression.

In conclusion, the steroid hormones including E2-17β and P4 are key regulators of many physiological processes and have been the subject of extensive previous research, particularly in classical steroid responsive tissues such as the uterus, mammary gland, and ovary. However, evaluation of E2-17β and P4 action in the liver, not generally regarded as a tissue that is regulated by steroid hormone, is particularly pertinent given the critical physiological role of the liver in regulation of energy homeostasis and metabolism of numerous substances including the steroid hormones. Steroid metabolism is extremely elevated in lactating cows and this elevated steroid metabolism underlies many of the changes in reproductive physiology and fertility that have been found in these animals [24]. Therefore we felt that understanding the effects of E2-17β and P4 on hepatic gene expression could provide insight into both the basic biology of E2-17β and P4 action and could provide additional insights into links between hepatic function and reproduction in dairy cattle. Collectively, the present study demonstrated for the first time a remarkable similarity in action for physiological concentrations of E2-17β and P4 in regulation of gene expression in the liver. There was little indication that these two distinct hormones elicited antagonistic or synergistic actions in the liver. Indeed most regulated genes were regulated similarly in magnitude and direction by E2T, P4T, and E2P4T. This uncommon pattern of gene expression in response to steroid hormones is consistent with the hypothesis that there are tissue-specific responses to steroid hormones. Thus classical steroid-responsive tissues may have dramatic and steroid-specific responses to E2-17β and P4, whereas, other tissues, such as the liver, may have changes in gene expression in response to steroid hormones but these changes may not be steroid specific and therefore not dramatically different during different of the stages of the estrous or menstrual cycle. Given the central role of the liver in regulating numerous metabolic functions including metabolism and deactivation of steroid hormones, this result may indicate that hepatic homeostasis can be maintained regardless of phase of the cycle by the activation of common biological pathways by distinct steroid hormones. These findings may also have important functional implications for hepatic gene expression in women or animals at different stages of the ovarian cycle or during treatments with different steroid hormones during birth control or hormone replacement.

## Supporting Information

Table S1 Top 40 genes in the bovine liver that were differentially expressed in response to estradiol.
(DOC)

Table S2 Top 40 genes in the bovine liver that were differentially expressed in response to progesterone.
(DOC)

Table S3 Top 40 genes in the bovine liver that were differentially expressed in response to estradiol+progesterone treatment.
(DOC)
