# Supplementary material for: Estradiol and Progesterone Exhibit Similar Patterns of Hepatic Gene Expression Regulation in the Bovine Model
Source: PLoS One. 2013 Sep 17;8(9):e73552. doi: 10.1371/journal.pone.0073552 (PMC3775788; doi:10.1371/journal.pone.0073552)
Supplement: Table S1 — Top 40 genes in the bovine liver that were differentially expressed in response to estradiol. (DOC) [file pone.0073552.s001.doc]

**Table S1.** Top 40 genes in the bovine liver that were differentially expressed in response to estradiol.

| **Gene** | **Gene Description** | **Gene function** | **Estradiol** | | **Progesterone** | | **Estradiol + Progesterone** | |
| --- | --- | --- | --- | --- | --- | --- | --- | --- |
|  |  |  | **Fold-Change** | ***P*** | **Fold-Change** | ***P*** | **Fold-Change** | ***P*** |
| **Upregulated Genes** | |  |  |  |  |  |  |  |
| *SECTM1* | secreted and transmembrane 1 | --- | 5.6 | 4.6E-05 | 4.0 | 4.8E-04 | 2.4 | 1.2E-02 |
| *PPP2R5E* | protein phosphatase 2, regulatory subunit B', epsilon isoform | binding, protein phosphatase type 2A regulator activity | 3.9 | 1.4E-03 | 2.7 | 1.4E-02 | 3.6 | 3.0E-03 |
| *EFEMP2* | EGF-containing fibulin-like extracellular matrix protein 2 | transmembrane receptor activity, calcium ion binding | 3.5 | 2.0E-03 | 1.9 | 7.1E-02 | -1.1 | 7.7E-01 |
| *CDK2* | cyclin-dependent kinase 2 | nucleotide binding, transferase activity, protein kinase activity | 2.5 | 2.2E-03 | 2.0 | 2.1E-02 | 3.0 | 1.0E-03 |
| *---* | strongly similar to insulin receptor substrate-1 isoform 1 [Bos taurus] | --- | 2.4 | 1.1E-03 | 2.3 | 2.0E-03 | 2.4 | 1.5E-03 |
| *MGC165862* | hypothetical LOC614805 | --- | 2.2 | 8.9E-05 | 2.0 | 4.2E-04 | 1.5 | 2.1E-02 |
| *C3orf57* | hypothetical protein LOC780785 | --- | 1.9 | 8.7E-03 | 2.3 | 3.1E-03 | 2.2 | 4.2E-03 |
| *C24H18ORF10* | chromosome 18 open reading frame 10 ortholog | --- | 1.9 | 9.6E-03 | 2.1 | 6.1E-03 | 2.3 | 2.6E-03 |
| *GLDC* | Glycine dehydrogenase (decarboxylating) | --- | 1.9 | 2.5E-04 | 1.4 | 3.5E-02 | 1.6 | 2.7E-03 |
| *UQCRB* | Ubiquinol-cytochrome c reductase binding protein | ubiquinol-cytochrome-c reductase activity | 1.8 | 1.3E-03 | 1.8 | 1.9E-03 | 1.5 | 1.6E-02 |
| *COX6A1* | cytochrome c oxidase subunit VIa polypeptide 1 | cytochrome-c oxidase activity, electron carrier activity | 1.8 | 1.7E-03 | 1.7 | 7.6E-03 | 1.3 | 1.1E-01 |
| *TPRKB* | TP53RK binding protein | --- | 1.8 | 1.6E-03 | 1.6 | 7.0E-03 | 1.8 | 1.6E-03 |
| *C5H12orf32* | chromosome 12 open reading frame 32 ortholog | --- | 1.8 | 1.0E-03 | 1.9 | 7.1E-04 | 1.5 | 1.1E-02 |
| *GNG5* | guanine nucleotide binding protein (G protein), gamma 5 | signal transducer activity | 1.7 | 2.2E-03 | 1.6 | 7.9E-03 | 1.6 | 1.2E-02 |
| *SH3BGRL2* | SH3 domain binding glutamic acid-rich protein like 2 | --- | 1.7 | 6.5E-04 | 1.5 | 8.4E-03 | 1.6 | 2.3E-03 |
| *B4GALT1* | UDP-Gal:betaGlcNAc beta 1,4- galactosyltransferase, polypeptide 1 | UDP-galactosyltransferase activity, metal ion binding | 1.7 | 1.2E-03 | 1.4 | 1.8E-02 | 1.4 | 2.5E-02 |
| *DHX40* | DEAH (Asp-Glu-Ala-His) box polypeptide 40 | nucleotide binding, hydrolase activity | 1.7 | 6.5E-04 | 1.4 | 1.4E-02 | 1.4 | 1.0E-02 |
| *PPIA* | peptidylprolyl isomerase A (cyclophilin A) | peptidyl-prolyl cis-trans isomerase activity, peptide binding | 1.7 | 1.7E-03 | 1.5 | 7.9E-03 | 1.7 | 2.7E-03 |
| *ELOVL5* | ELOVL family member 5, elongation of long chain fatty acids (FEN1/Elo2, SUR4/Elo3-like, yeast) | fatty acid biosynthetic process, lipid biosynthetic process | 1.7 | 5.6E-05 | 1.3 | 1.9E-02 | 1.3 | 2.4E-02 |
| *PDE6C* | phosphodiesterase 6C, cGMP-specific, cone, alpha prime | catalytic activity, 3',5'-cyclic-nucleotide phosphodiesterase activity | 1.6 | 1.9E-03 | 1.3 | 8.3E-02 | 1.6 | 4.4E-03 |
| ***Downregulated*** |  |  |  |  |  |  |  |  |
| *MYPN* | myopalladin | --- | -10.6 | 6.2E-04 | -3.7 | 3.3E-02 | -2.9 | 7.0E-02 |
| *GPR155* | G protein-coupled receptor 155 | --- | -9.4 | 5.9E-04 | -2.2 | 1.5E-01 | -2.0 | 2.1E-01 |
| *FAM80B* | family with sequence similarity 80, member B | nucleotide binding, metal ion binding | -6.1 | 9.4E-03 | -11.9 | 1.7E-03 | -2.9 | 1.1E-01 |
| *LOC540169* | Hypothetical LOC540169 | --- | -5.8 | 9.6E-04 | -2.5 | 5.1E-02 | -2.7 | 4.0E-02 |
| *ATR* | Ataxia telangiectasia and Rad3 related | --- | -5.6 | 3.9E-04 | -2.2 | 5.6E-02 | -3.2 | 7.9E-03 |
| *KDR* | kinase insert domain receptor (a type III receptor tyrosine kinase) | nucleotide binding, transferase activity | -5.2 | 8.0E-04 | -2.7 | 2.8E-02 | -1.4 | 4.1E-01 |
| *LOC511442* | hypothetical LOC511442 | nucleus | -4.3 | 3.9E-03 | -4.8 | 3.2E-03 | -1.3 | 5.2E-01 |
| *H18C16ORF77* | Chromosome 16 open reading frame 77 ortholog | --- | -4.2 | 3.4E-03 | -1.8 | 1.9E-01 | -4.2 | 4.6E-03 |
| *COL2A1* | collagen, type II, alpha 1 | structural molecule activity, extracellular matrix structural constituent | -4.1 | 1.1E-03 | -1.9 | 8.7E-02 | -1.7 | 1.7E-01 |
| *RHOB* | Ras homolog gene family, member B | nucleotide binding, protein binding | -4.0 | 1.9E-04 | -2.0 | 2.6E-02 | -1.1 | 7.4E-01 |
| *PPARD* | peroxisome proliferator-activated receptor delta | DNA binding, transcription factor activity, steroid hormone receptor activity | -3.9 | 9.3E-04 | -1.9 | 6.6E-02 | -2.0 | 5.0E-02 |
| *CSPG5* | chondroitin sulfate proteoglycan 5 -neuroglycan C | --- | -3.9 | 1.0E-03 | -1.7 | 1.4E-01 | -2.2 | 3.1E-02 |
| *ADRB3* | adrenergic, beta-3-, receptor | rhodopsin-like receptor activity, signal transducer activity, receptor activity | -3.7 | 7.5E-05 | -3.9 | 8.2E-05 | -2.8 | 8.3E-04 |
| *CORO2A* | coronin, actin binding protein, 2A | actin binding | -3.5 | 1.9E-04 | -2.7 | 2.1E-03 | -1.9 | 2.7E-02 |
| *AP4M1* | adaptor-related protein complex 4, mu 1 subunit | protein binding, protein transporter activity | -3.5 | 1.2E-04 | -2.6 | 1.7E-03 | -2.5 | 2.5E-03 |
| *RBM23* | RNA binding motif protein 23 | nucleic acid binding, RNA binding | -3.4 | 1.8E-02 | -1.2 | 7.5E-01 | -6.0 | 2.6E-03 |
| *---* | TL strongly similar to antagonizer of myc transcriptional activity-1 | --- | -3.2 | 3.0E-05 | -3.8 | 1.3E-05 | -1.8 | 8.1E-03 |
| *MXRA5* | Matrix-remodelling associated 5 | protein binding | -3.0 | 1.8E-03 | -1.8 | 5.8E-02 | -2.6 | 5.8E-03 |
| *TRPC2* | transient receptor potential channel 2 | damaged DNA binding, ion channel activity | -2.9 | 3.2E-02 | -7.5 | 1.1E-03 | -3.8 | 1.5E-02 |
| *LAD1* | ladinin 1 | --- | -2.9 | 1.5E-02 | -8.0 | 1.7E-04 | -1.5 | 3.3E-01 |
