# Supplementary material for: Estradiol and Progesterone Exhibit Similar Patterns of Hepatic Gene Expression Regulation in the Bovine Model
Source: PLoS One. 2013 Sep 17;8(9):e73552. doi: 10.1371/journal.pone.0073552 (PMC3775788; doi:10.1371/journal.pone.0073552)
Supplement: Table S2 — Top 40 genes in the bovine liver that were differentially expressed in response to progesterone. (DOC) [file pone.0073552.s002.doc]

**Table S2**. Top 40 genes in the bovine liver that were differentially expressed in response to progesterone.

| **Gene** | **Gene Description** | **Gene function** | **Estradiol** | | **Progesterone** | | **Estradiol + Progesterone** | |
| --- | --- | --- | --- | --- | --- | --- | --- | --- |
|  |  |  | **Fold-Change** | ***P*** | **Fold-Change** | ***P*** | **Fold-Change** | ***P*** |
| **Upregulated Genes** | |  |  |  |  |  |  |  |
| *SECTM1* | secreted and transmembrane 1 | --- | 5.6 | 4.6E-05 | 4.0 | 4.8E-04 | 2.4 | 1.2E-02 |
| *PPP2R5E* | protein phosphatase 2, regulatory subunit B', epsilon isoform | binding, protein phosphatase type 2A regulator activity | 3.9 | 1.4E-03 | 2.7 | 1.4E-02 | 3.6 | 3.0E-03 |
| *---* | Transcribed locus, strongly similar to XP_581382.2 PREDICTED: similar to insulin receptor substrate-1 isoform 1 [Bos taurus] | --- | 2.4 | 1.1E-03 | 2.3 | 2.0E-03 | 2.4 | 1.5E-03 |
| *C24H18ORF10* | chromosome 18 open reading frame 10 ortholog | --- | 1.9 | 9.6E-03 | 2.1 | 6.1E-03 | 2.3 | 2.6E-03 |
| *CDK2* | cyclin-dependent kinase 2 | nucleotide binding, protein kinase activity | 2.5 | 2.2E-03 | 2.0 | 2.1E-02 | 3.0 | 1.0E-03 |
| *C5H12orf32* | chromosome 12 open reading frame 32 ortholog | --- | 1.8 | 1.0E-03 | 1.9 | 7.1E-04 | 1.5 | 1.1E-02 |
| *UQCRB* | Ubiquinol-cytochrome c reductase binding protein | ubiquinol-cytochrome-c reductase activity | 1.8 | 1.3E-03 | 1.8 | 1.9E-03 | 1.5 | 1.6E-02 |
| *AK3* | adenylate kinase 3 | adenylate kinase activity, ATP binding | 1.6 | 6.5E-03 | 1.7 | 4.5E-03 | 1.7 | 4.6E-03 |
| *COX6A1* | cytochrome c oxidase subunit VIa polypeptide 1 | cytochrome-c oxidase activity, electron carrier activity | 1.8 | 1.7E-03 | 1.7 | 7.6E-03 | 1.3 | 1.1E-01 |
| *TPRKB* | TP53RK binding protein | --- | 1.8 | 1.6E-03 | 1.6 | 7.0E-03 | 1.8 | 1.6E-03 |
| *GNG5* | guanine nucleotide binding protein (G protein), gamma 5 | signal transducer activity | 1.7 | 2.2E-03 | 1.6 | 7.9E-03 | 1.6 | 1.2E-02 |
| *ATP5G3* | ATP synthase, H+ transporting, mitochondrial F0 complex, subunit C3 (subunit 9) | ATP metabolic process, protein and lipid binding | 1.6 | 9.4E-05 | 1.6 | 1.4E-04 | 1.5 | 2.5E-04 |
| *BDH2* | 3-hydroxybutyrate dehydrogenase, type 2 | fatty acid beta-oxidation, catalytic activity, 3-hydroxybutyrate dehydrogenase activity, oxidoreductase activity | 1.6 | 3.4E-03 | 1.6 | 8.1E-03 | 1.7 | 3.0E-03 |
| *PPIA* | peptidylprolyl isomerase A (cyclophilin A) | peptidyl-prolyl cis-trans isomerase activity, peptide binding | 1.7 | 1.7E-03 | 1.5 | 7.9E-03 | 1.7 | 2.7E-03 |
| *SH3BGRL2* | SH3 domain binding glutamic acid-rich protein like 2 | --- | 1.7 | 6.5E-04 | 1.5 | 8.4E-03 | 1.6 | 2.3E-03 |
| *B4GALT1* | UDP-Gal:betaGlcNAc beta 1,4- galactosyltransferase, polypeptide 1 | epithelial cell development, lactose synthase activity, UDP-galactosyltransferase activity, metal ion binding | 1.7 | 1.2E-03 | 1.4 | 1.8E-02 | 1.4 | 2.5E-02 |
| *DHX40* | DEAH (Asp-Glu-Ala-His) box polypeptide 40 | nucleic acid binding, helicase activity, ATP binding | 1.7 | 6.5E-04 | 1.4 | 1.4E-02 | 1.4 | 1.0E-02 |
| *NDUFC1* | NADH dehydrogenase (ubiquinone) 1, subcomplex unknown, 1, 6kDa | transport, oxidation reduction | 1.6 | 2.1E-06 | 1.4 | 1.7E-04 | 1.3 | 4.8E-04 |
| *GLDC* | Glycine dehydrogenase (decarboxylating) | --- | 1.9 | 2.5E-04 | 1.4 | 3.5E-02 | 1.6 | 2.7E-03 |
| **Downregulated** |  |  |  |  |  |  |  |  |
| *FAM80B* | family with sequence similarity 80, member B | protein modification process, nucleotide binding, ATP and ion binding | -6.1 | 9.4E-03 | -11.9 | 1.7E-03 | -2.9 | 1.1E-01 |
| *LAD1* | ladinin 1 | --- | -2.9 | 1.5E-02 | -8.0 | 1.7E-04 | -1.5 | 3.3E-01 |
| *TRPC2* | transient receptor potential channel 2 | damaged DNA binding, ion (calcium) channel activity | -2.9 | 3.2E-02 | -7.5 | 1.1E-03 | -3.8 | 1.5E-02 |
| *NAP1L4* | Nucleosome assembly protein 1-like 4 | nucleus | -2.8 | 1.3E-02 | -5.8 | 4.1E-04 | -3.5 | 4.6E-03 |
| *LOC511442* | hypothetical LOC511442 | --- | -4.3 | 3.9E-03 | -4.8 | 3.2E-03 | -1.3 | 5.2E-01 |
| *ADRB3* | adrenergic, beta-3-, receptor | signal transduction, G-protein coupled receptor activity | -3.7 | 7.5E-05 | -3.9 | 8.2E-05 | -2.8 | 8.3E-04 |
| *---* | TL, strongly similar to antagonizer of myc transcriptional activity-1 | --- | -3.2 | 3.0E-05 | -3.8 | 1.3E-05 | -1.8 | 8.1E-03 |
| *MYPN* | myopalladin | --- | -10.6 | 6.2E-04 | -3.7 | 3.3E-02 | -2.9 | 7.0E-02 |
| *CCDC97* | Coiled-coil domain containing 97 | --- | -2.8 | 8.3E-03 | -3.0 | 7.3E-03 | -3.6 | 2.9E-03 |
| *CENTB1* | centaurin, beta 1 | signal transducer activity, zinc ion binding, hydrolase activity | -2.9 | 1.6E-03 | -2.7 | 3.5E-03 | -1.8 | 6.5E-02 |
| *CORO2A* | coronin, actin binding protein, 2A | actin binding | -3.5 | 1.9E-04 | -2.7 | 2.1E-03 | -1.9 | 2.7E-02 |
| *KDR* | kinase insert domain receptor (a type III receptor tyrosine kinase) | nucleotide binding, protein kinase activity | -5.2 | 8.0E-04 | -2.7 | 2.8E-02 | -1.4 | 4.1E-01 |
| *AP4M1* | adaptor-related protein complex 4, mu 1 subunit | protein complex assembly, transport, protein binding | -3.5 | 1.2E-04 | -2.6 | 1.7E-03 | -2.5 | 2.5E-03 |
| *TRA@* | T cell receptor, alpha | receptor activity | -2.9 | 2.2E-03 | -2.3 | 1.3E-02 | 1.1 | 7.3E-01 |
| *MYOC* | myocilin, trabecular meshwork inducible glucocorticoid response | protein binding, latrotoxin receptor activity | -2.6 | 3.0E-04 | -2.2 | 2.2E-03 | -2.0 | 5.0E-03 |
| *SALL2* | sal-like 2 (Drosophila) | --- | -2.9 | 7.8E-04 | -2.1 | 1.1E-02 | -2.7 | 1.8E-03 |
| *WNT2B* | wingless-type MMTV integration site family, member 2B | signal transducer activity | -2.6 | 8.5E-04 | -2.1 | 7.9E-03 | -2.0 | 8.2E-03 |
| *RHOB* | Ras homolog gene family, member B | nucleotide binding, protein binding | -4.0 | 1.9E-04 | -2.0 | 2.6E-02 | -1.1 | 7.4E-01 |
| *C1QTNF6* | C1q and tumor necrosis factor related protein 6 | extracellular region | -2.7 | 1.1E-03 | -1.9 | 2.2E-02 | -1.3 | 3.8E-01 |
| *DEPDC6* | DEP domain containing 6 | protein binding | -2.7 | 4.8E-04 | -1.8 | 2.3E-02 | -1.8 | 2.2E-02 |
