# Supplementary material for: Estradiol and Progesterone Exhibit Similar Patterns of Hepatic Gene Expression Regulation in the Bovine Model
Source: PLoS One. 2013 Sep 17;8(9):e73552. doi: 10.1371/journal.pone.0073552 (PMC3775788; doi:10.1371/journal.pone.0073552)
Supplement: Table S3 — Top 40 genes in the bovine liver that were differentially expressed in response to estradiol+progesterone treatment. (DOC) [file pone.0073552.s003.doc]

**Table S3**. Top 40 genes in the bovine liver that were differentially expressed in response to estradiol + progesterone treatment.

| **Gene** | **Gene Description** | **Gene function** | **Estradiol** | | | | **Progesterone** | | | **Estradiol + Progesterone** | |
| --- | --- | --- | --- | --- | --- | --- | --- | --- | --- | --- | --- |
|  |  |  | **Fold-Change** | | | ***P*** | **Fold-Change** | | ***P*** | **Fold-Change** | ***P*** |
| **Upregulated genes** | |  |  | | |  |  | |  |  |  |
| *PPP2R5E* | protein phosphatase 2, regulatory subunit B', epsilon isoform | signal transduction, protein phosphatase type 2A complex | 3.9 | | | 1.4E-03 | 2.7 | | 1.4E-02 | 3.6 | 3.0E-03 |
| *CDK2* | cyclin-dependent kinase 2 | protein amino acid phosphorylation, cell cycle, mitosis | 2.5 | | | 2.2E-03 | 2.0 | | 2.1E-02 | 3.0 | 1.0E-03 |
| *SECTM1* | secreted and transmembrane 1 | --- | 5.6 | | | 4.6E-05 | 4.0 | | 4.8E-04 | 2.4 | 1.2E-02 |
| *C24H18ORF10* | chromosome 18 open reading frame 10 ortholog | --- | 1.9 | | | 9.6E-03 | 2.1 | | 6.1E-03 | 2.3 | 2.6E-03 |
| *TMED6* | transmembrane emp24 protein transport domain containing 6 | transport | 1.6 | | | 4.7E-02 | 2.9 | | 2.9E-04 | 2.1 | 3.8E-03 |
| *TPRKB* | TP53RK binding protein | --- | 1.8 | | | 1.6E-03 | 1.6 | | 7.0E-03 | 1.8 | 1.6E-03 |
| *FAM108B1* | Family with sequence similarity 108, member B1 | --- | 1.5 | | | 4.9E-03 | 1.9 | | 5.2E-04 | 1.7 | 1.4E-03 |
| *BDH2* | 3-hydroxybutyrate dehydrogenase, type 2 | fatty acid beta-oxidation, metabolic process, oxidoreductase activity | 1.6 | | | 3.4E-03 | 1.6 | | 8.1E-03 | 1.7 | 3.0E-03 |
| *TRAK2* | Trafficking protein, kinesin binding 2 | --- | 1.5 | | | 1.5E-03 | 1.1 | | 3.0E-01 | 1.7 | 5.3E-04 |
| *AK3* | adenylate kinase 3 | nucleobase, nucleoside, nucleotide and nucleic acid metabolic process | 1.6 | | | 6.5E-03 | 1.7 | | 4.5E-03 | 1.7 | 4.6E-03 |
| *SEP7* | septin 7 | cell cycle, nucleotide binding, protein binding | 1.5 | | | 1.2E-02 | 1.6 | | 4.5E-03 | 1.7 | 2.4E-03 |
| *PPIA* | peptidylprolyl isomerase A (cyclophilin A) | protein folding, isomerase activity | 1.7 | | | 1.7E-03 | 1.5 | | 7.9E-03 | 1.7 | 2.7E-03 |
| *GLDC* | Glycine dehydrogenase (decarboxylating) | --- | 1.9 | | | 2.5E-04 | 1.4 | | 3.5E-02 | 1.6 | 2.7E-03 |
| *SH3BGRL2* | SH3 domain binding glutamic acid-rich protein like 2 | --- | 1.7 | | | 6.5E-04 | 1.5 | | 8.4E-03 | 1.6 | 2.3E-03 |
| *PDE6C* | phosphodiesterase 6C, cGMP-specific, cone, alpha prime | signal transduction, visual perception, hydrolase activity | 1.6 | | | 1.9E-03 | 1.3 | | 8.3E-02 | 1.6 | 4.4E-03 |
| *TSPAN7* | tetraspanin 7 | dicarboxylic acid transport | 1.5 | | | 3.2E-03 | 1.7 | | 7.4E-04 | 1.6 | 2.3E-03 |
| *NDUFB6* | NADH dehydrogenase (ubiquinone) 1 beta subcomplex, 6, 17kDa | transport, oxidation reduction | 1.6 | | | 1.1E-03 | 1.7 | | 3.8E-04 | 1.6 | 1.1E-03 |
| *OTC* | ornithine carbamoyltransferase | urea cycle, amino acid metabolic process | 1.4 | | | 1.7E-02 | 1.6 | | 2.5E-03 | 1.6 | 2.8E-03 |
| *TOMM20* | translocase of outer mitochondrial membrane 20 homolog (yeast) | protein targeting, intracellular protein transport, protein transport | 1.5 | | | 2.1E-03 | 1.6 | | 1.3E-03 | 1.6 | 1.5E-03 |
| *GNG5* | guanine nucleotide binding protein (G protein), gamma 5 | signal transduction, G-protein coupled receptor protein signaling pathway | 1.7 | | | 2.2E-03 | 1.6 | | 7.9E-03 | 1.6 | 1.2E-02 |
| **Downregulated genes** | | | | | | | | | | | |
| *RBM23* | RNA binding motif protein 23 | mRNA processing, nucleotide bindinG | | -3.4 | 1.8E-02 | | | -1.2 | 7.5E-01 | -6.0 | 2.6E-03 |
| *KCNJ15* | potassium inwardly-rectifying channel, subfamily J, member 15 | transport, ion transport, potassium ion transport | | -1.5 | 2.5E-01 | | | -5.7 | 2.7E-04 | -5.0 | 5.2E-04 |
| *LYST* | lysosomal trafficking regulator | transport, lysosome organization and biogenesis, protein transport | | -1.2 | 7.2E-01 | | | -8.2 | 3.5E-03 | -4.3 | 2.7E-02 |
| *H18C16ORF77* | Chromosome 16 open reading frame 77 ortholog | --- | | -4.2 | 3.4E-03 | | | -1.8 | 1.9E-01 | -4.2 | 4.6E-03 |
| *LOC510844* | Similar to Alpha-fetoprotein enhancer-binding protein (AT motif-binding factor) (AT-binding transcription factor 1) | --- | | -1.5 | 2.9E-01 | | | -2.5 | 2.2E-02 | -4.2 | 1.6E-03 |
| *NCOR1* | nuclear receptor co-repressor 1 | regulation of transcription, DNA binding | | -1.8 | 1.1E-01 | | | -3.3 | 5.4E-03 | -4.1 | 1.8E-03 |
| *MED13L* | mediator complex subunit 13-like | --- | | -2.4 | 1.8E-02 | | | -1.4 | 3.9E-01 | -4.0 | 1.5E-03 |
| *INSR* | insulin receptor | proteolysis | | -1.5 | 1.8E-01 | | | -1.8 | 7.0E-02 | -4.0 | 7.0E-04 |
| *TRPC2* | transient receptor potential channel 2 | single strand break repair, ion transport | | -2.9 | 3.2E-02 | | | -7.5 | 1.1E-03 | -3.8 | 1.5E-02 |
| *TMEM32* | Transmembrane protein 32 | integral to membrane | | -1.7 | 8.5E-02 | | | -2.2 | 2.3E-02 | -3.8 | 8.4E-04 |
| *EHBP1L1* | EH domain binding protein 1-like 1 | --- | | -2.1 | 2.5E-02 | | | -2.9 | 4.5E-03 | -3.7 | 1.1E-03 |
| *CCDC97* | Coiled-coil domain containing 97 | --- | | -2.8 | 8.3E-03 | | | -3.0 | 7.3E-03 | -3.6 | 2.9E-03 |
| *NAP1L4* | Nucleosome assembly protein 1-like 4 | nucleosome assembly | | -2.8 | 1.3E-02 | | | -5.8 | 4.1E-04 | -3.5 | 4.6E-03 |
| *HBB /// HBE1* | hemoglobin, beta /// hemoglobin, epsilon 1 | transport, oxygen transport | | -2.3 | 3.4E-03 | | | -2.2 | 6.4E-03 | -3.4 | 2.5E-04 |
| *CCDC136* | coiled-coil domain containing 136 | --- | | -2.3 | 1.6E-02 | | | -3.0 | 4.1E-03 | -3.4 | 2.0E-03 |
| *PAK1* | P21/Cdc42/Rac1-activated kinase 1 (STE20 homolog, yeast) | protein amino acid phosphorylation | | -2.0 | 2.1E-02 | | | -1.7 | 8.1E-02 | -3.3 | 1.1E-03 |
| *ATN1* | atrophin 1 | --- | | -1.3 | 4.3E-01 | | | -1.2 | 6.2E-01 | -3.2 | 2.0E-03 |
| *ATR* | Ataxia telangiectasia and Rad3 related | --- | | -5.6 | 3.9E-04 | | | -2.2 | 5.6E-02 | -3.2 | 7.9E-03 |
| *P2RX7* | purinergic receptor P2X, ligand-gated ion channel, 7 | transport, ion transport | | -2.5 | 4.2E-03 | | | -2.2 | 1.3E-02 | -3.1 | 1.1E-03 |
| *ADRB3* | adrenergic, beta-3-, receptor | signal transduction, activation of adenylate cyclase activity | | -3.7 | 7.5E-05 | | | -3.9 | 8.2E-05 | -2.8 | 8.3E-04 |
